# Supplementary material for: Learning curve and surgical outcome of robotic assisted colorectal surgery with ERAS program
Source: Sci Rep. 2022 Nov 29;12:20566. doi: 10.1038/s41598-022-24665-w (PMC9709162; doi:10.1038/s41598-022-24665-w)
Supplement: Supplementary file 2 — Supplementary Information 2. [file 41598_2022_24665_MOESM2_ESM.zip › RAL Raw data-Ñ[▒K/102/HS_ñw╝╨░O▒Kñσ.pdf]

正式摘要(電子簽章：000000222103043)		
W83 / W83 ☐ 英文標題(注意:無英文姓名-請補病人基本檔!)	☐【讀取】憑證	

臺中榮民總醫院
出院病歷摘要

轉入號： 轉出號：

[redacted]

(8)流水編號	
(9)入院日期	110/07/28 (2021) 直腸外科 W83 - 083
(10)轉科日期	
(11)出院日期	110/08/02 (2021) 住院天數計 5 日

診斷

- 入院：1. Rectal adenocarcinoma, 10cm FAV, 4.9 cm in diameter, s/p tissue proof at Lin-Shin hospital, with tumor invading non-peritonealized pericolonic or perirectal tissuess,
  2. Right upper-third ureteral stone with marked hydronephrosis
- 出院：1. Rectal adenocarcinoma, 10cm FAV, 4.9 cm in diameter, with tumor invading non-peritonealized pericolonic or perirectal tissuess
  -s/p Robotic LAR on 2021/07/29
  2. Right upper-third ureteral stone with marked hydronephrosis
  -s/p Rt ureterolithotomy + 6*24 DJ insertion on 2021/7/29

主訴

- loose stool for two months, small caliber, with intermittent blood-tinged

病史

- This 67-year-old woman is a case of:
  1. Rectal adenocarcinoma, 10cm FAV, 4.9 cm in diameter, s/p tissue proof at Lin-Shin hospital, with tumor invading non-peritonealized pericolonic or perirectal tissues, r/o obstruction
  2. Right upper-third ureteral stone (2.1cm, Ser203-img14) with hydronephrosis, s/p ESWL for twice in her age 30-40s, without further follow up
  3. C/S for three times

  She presented with loose stool for two months, small caliber, with intermittent blood-tinged. Body weight loss was also noted with decreased appetite, dropped 7kg within 2 years, particularly 4kg in the past two months. There was no abdominal discomfort, nausea or vomiting, nor constipation.

  She visited Lin-shin hospital at first, where UGI/LGI scope were arranged. Colonscopic biopsy reported adenocarcinoma. She then started semi-liquid diet in the past one month, and still stool passage was noted. She then came to our hospital for further management. CEA was elevated as 21.58. Abdominal CT on 2021/07/21 reported a 4.9 cm rectal tumor, invading non-peritonealized pericolonic or perirectal tissues. Irregular wall thickening of the rectum was mentioned with increased perirectal strandings, compatible with rectal cancer. Besides, right upper-third ureteral stone(2.1cm,Ser203-img14) is mentioned with marked right hydronephrosis. PET on 2021/07/23 reported rectal cancer (Grade 4), as well as small lesions over bilateral lung fields (Grade 2, inflammation, r/o tumor involvement). After discussion, the patient decided to receive Robotic LAR.

體檢發現

-
  - General appearance :
    - easy looking , conscious clear , without cardiopulmonary distress
    - Vital signs自動帶入date 體溫(BT) 脈搏(P) 呼吸(R) 血壓(BP) 身高(BH) 體重(BW) BMI
      20210728 36.5℃ 91次/分 18次/分 142/75mmHg 147cm 41kg 19.0
    - Vital signs: B.P. 142/75 mmHg , P.R. 91 /min , R.R. 18 /min , B.T. 36.5 ℃
  - HEENT :
    - Eyes: no anemic conjunctiva, no icteric sclera, normal EOM
    - ENT: no otorrhea, no rhinorrhea, no erythematous mucosa, no injected tonsil, no deformity
  - Neck: supple, freely movable, trachea midline,no deformity, no engorged jugular vein , no palpable thyroid or lymph node enlargement

-
  - Chest:
    - Inspection: Grossly normal, symmetric and fully expansion, no accessory muscle used, no operation scar, no spider angiomata, no gynecomastia
    - Palpation: no palpable mass or nodule, no subcutaneous emphysema, no tenderness
    - Ausculation: clear breathing sound
  - Heart:
    - Palpation: no thrill, no heave
    - Percussion: normal heart size
    - Ausculation: regular heart beat, normal S1, S2, no gallop, no murmur
  - Extremities: No deformity, full ranged of motion without limitation, no clubbing finger or nail cyanosis, no pitting edema, no ulcer or pigmentation, no obvious skin lesion, no varicose vein, no bone, joint or muscle tenderness

CRS CONDITION

- Karnofsky performance status: (100) Normal, no complaints, no evidence of disease
- PE of Abdomen:
- __1. Inspection: flat
- __3. Palapation: soft, without tenderness
- CEA: 21.58 ng/ml
- Imaging Studies:
- Colonoscope report: Colonscopic biopsy reported adenocarcinoma, 10cm FAV. (from Lin-Shin hospital)
- Sono , CT of abdomen and chest Films:Abdominal CT on 2021/07/21 reported a 4.9 cm rectal tumor, invading non-peritonealized pericolonic or perirectal tissues. Irregular wall thickening of the rectum was mentioned with increased perirectal strandings, compatible with rectal cancer. Besides, right upper-third ureteral stone(2.1cm,Ser203-img14) is mentioned with marked right hydronephrosis.
  PET on 2021/07/23 reported rectal cancer (Grade 4), as well as small lesions over bilateral lung fields (Grade 2, inflammation, r/o tumor involvement).
- Figures:
  1. colon & rectum

手術日期及方法

- 手術日期及方法20210729 Robot Assisted Low Anterior Resection
  20210729 robotic assisted Rt ureterolithotomy + 6*24 DJ insertion
- 手術結果及發現：
  - 1. Rectal tumor about 3x3 10 cm in size at 8cm FAV with local advanced. 2. LAR was complet 3. IMA high ligation+ 4. distal margin 2 cm, Surefrom 45 *2 for rectum 5. air tie test neg. donut intact. penrose in right ureter.

住院治療經過

- After admission, we performed the routine serum survey and preoperative prepare based on ERAS protocol. She underwent robotic assisted low anterior resection and right ureterolithotomy with D-J insertion on 2021/07/29. After surgery, she tolerated well, presented with fair oral intake and smooth self-voiding. Penrose drain was removed. GU doctor suggested keeping D-J catheter for one month. Under her relative stable condition, she was discharged on 08/02 and arranged OPD follow-up.

合併症

一般檢查

- 放射線報告 :檢驗項目 **:ABDOMEN (KUB),SUPINE** 報告日期 **:1100803**
  主　訴：
  Result:N
  Finding:
  > Some bowel gas and fecal material are noted.
  > DJD and scoliosis of T-L spine with marginal spur formation
  > S/P double-J implantation in right collecting system
  > Surgical clips retention over right upper abdomen region, and pelvis region
  > On drainage tube over right abdominal region
  > Some phleboliths over pelvic area are noted

- > Subcutaneous emphysema over bilateral flank, r/o post operation change. Suggest follow-up.
  Impression:
  As above description.
- **檢驗項目 :Bilirubin Total,Bilirubin Direct 檢體:Blood 報告日期 :1100729**
  Total Bilirubin 0.58 mg/dL (0.2-1.2)
  Direct Bilirubin 0.18 mg/dL (=0.2)(自106年10月20日起修訂Direct Bilirubin參考值)
  備註:
  Remark :

  檢驗項目 :BUN,Creatinine,Na,K,Cl,Ca 檢體:Blood 報告日期 :1100729
  BUN 9 mg/dL (5- 25)
  Creatinine 0.64 mg/dL (M:0.7-1.4 , F:0.5-0.9)
  Na 142 mEq/L (137-153)
  K 3.6 mEq/L (3.5-5.3)
  Cl 108 mEq/L (95-105)
  Ca 7.4 mg/dL (8.4-10.2)
  eGFR 98.38 ml/min/1.73㎡ (STAGE1: >=90 ; STAGE2: 60-89 ;
  STAGE3: 30-59.9 ; STAGE4: 15-30 ; STAGE5: 15)
  備註:
  Remark :

  檢驗項目 :GOT,GPT,Albumin,ALK-PTASE 檢體:Blood 報告日期 :1100729
  AST(SGOT) 17 U/L (8 - 38)
  ALT(SGPT) 10 U/L (M:10-50,F:10-35)
  LDH U/L (120-240)
  ALP 59 U/L (50-190)
  Albumin 3.0 g/dL (3.5-5.0)
  Total Protein g/dL (6.0-8.0)
  FIB-4 1.757 (Metavir fibrosis stage F3 >= 3.25)
  備註:
  Remark :

  檢驗項目 :Glucose,Random 檢體:Blood 報告日期 :1100729
  Glucose 176 mg/dL (Random:70-200)
  備註:
  Remark :

  檢驗項目 :Differential Count(D.C),Platelet 檢體:Blood 報告日期 :1100729
  DC: SEG. % (40-74) BAND % (0-5) LYM 7.4 % (19-48)
  MONO. 3.8 % (3.4-9) EOSINO. 0.0 % (0-7) BASO. 0.2 % (0-1.5)
  META. % (0) MYELO. % (0) PROMYELO. % (0)
  BLAST. % (0) GRAN. % Atypical Cell %
  ATYP.LYM. % NEUT. 88.6 % (40-74)
  NRBC/100WBC. 0.0
  PLATELET COUNT. 205 x10^3/μL (150-400)
  備註:
  Remark :

特殊檢查

病理報告

- pending

其它

出院時情況

- 3.改門診治療

出院指示

藥名	劑量	劑量單位	用法	頻次	數量	數量單位	現況	天數	單位
(□)Takepron OD tab 30mg	1	TAB	PO	QDAC	7	TAB	TH	7	天
Mag. Oxide tab	2	TAB	PO	TID	42	TAB	TH	7	天

Ultracet tab	1	TAB	PO	BID	14	TAB	TH	7	天
Through tab	1	TAB	PO	QN	7	TAB	TH	7	天

出院計劃

- 門診日期
    - date on 2021/08/10

主治醫師 2062F 蔣鋒帆 / 住院醫師 2336L 洪浚嚴

正式摘要(電子簽章：**000000222103043)**

W83 / W83

doctorRank:B
